# Simulation-enhanced nurse mentoring to improve preeclampsia and eclampsia care: an education intervention study in Bihar, India

**DOI:** 10.1186/s12884-019-2186-x

**Published:** 2019-01-23

**Authors:** Julia H. Raney, Melissa C. Morgan, Amelia Christmas, Mona Sterling, Hilary Spindler, Rakesh Ghosh, Aboli Gore, Tanmay Mahapatra, Dilys M. Walker

**Affiliations:** 1 Yale School of Medicine, Yale University, 333 Cedar St, New Haven, CT 06510 USA; 2 Department of Pediatrics, University of California San Francisco, 550 16th Street, Box 1224, San Francisco, CA 94158 USA; 3 Maternal, Adolescent, Reproductive, and Child Health Centre, London School of Hygiene and Tropical Medicine, London, UK; 4 PRONTO International, State RMNCH+A Unit, C-16 Krishi Nagar, A.G. Colony, Patna, Bihar 80002 India; 5 Institute for Global Health Sciences, University of California San Francisco, 550 16th Street, Box 1224, San Francisco, CA 94158 USA; 6 CARE India, Bihar technical Support Program, 14, Patliputra Colony, Patna, Bihar 800013 India; 7 Department of Obstetrics and Gynecology and Reproductive Services, University of California San Francisco, 1001 Potrero Ave, San Francisco, CA 94110 USA

**Keywords:** Preeclampsia, Eclampsia, Simulation, Barriers, Enablers, India

## Abstract

**Background:**

Inadequately treated, preeclampsia and eclampsia (PE/E) may rapidly lead to severe complications in both mothers and neonates, and are estimated to cause 60,000 global maternal deaths annually. Simulation-based training on obstetric and neonatal emergency management has demonstrated promising results in low- and middle-income countries. However, the impact of simulation training on use of evidence-based practices for PE/E diagnosis and management in low-resource settings remains unknown.

**Methods:**

This study was based on a statewide, high fidelity in-situ simulation training program developed by PRONTO International and implemented in collaboration with CARE India on PE/E management in Bihar, India. Using a mixed methods approach, we evaluated changes over time in nurse mentees’ use of evidence-based practices during simulated births at primary health clinics. We compared the proportion and efficiency of evidence-based practices completed during nurse mentees’ first and last participation in simulated PE/E cases. Twelve semi-structured interviews with nurse mentors explored barriers and enablers to high quality PE/E care in Bihar.

**Results:**

A total of 39 matched first and last simulation videos, paired by facility, were analyzed. Videos occurred a median of 62 days apart and included 94 nurses from 33 primary health centers. Results showed significant increases in the median number of ‘key history questions asked,’ (1.0 to 2.0, *p* = 0.03) and ‘key management steps completed,’ (2.0 to 3.0, *p* = 0.03). The time from BP measured to magnesium sulfate given trended downwards by 3.2 min, though not significantly (*p* = 0.06). Key barriers to high quality PE/E care included knowledge gaps, resource shortages, staff hierarchy between physicians and nurses, and poor relationships with patients. Enablers included case-based and simulation learning, promotion of teamwork and communication, and effective leadership.

**Conclusion:**

Simulation training improved the use of evidence-based practices in PE/E simulated cases and has the potential to increase nurse competency in diagnosing and managing complex maternal complications such as PE/E. However, knowledge gaps, resource limitations, and interpersonal barriers must be addressed in order to improve care. Teamwork, communication, and leadership are key mechanisms to facilitate high quality PE/E care in Bihar.

## Background

Globally, an estimated 275,000 maternal deaths occurred in 2015 [1]. Hypertensive disorders of pregnancy, including preeclampsia and eclampsia (PE/E), are the second leading cause of maternal death in women under age 35 [1]. Mortality related to PE/E can be prevented with swift diagnosis, effective management, and timely delivery [2, 3]. However, evidence-based interventions are sparsely implemented in many low- and middle-income country settings, leading to poor outcomes for both mothers and neonates [4, 5].

In 2015, an estimated 64,000 maternal deaths occurred in India alone [1]. In 2005, the Government of India implemented Janani Suraksha Yojana (JSY), a nationwide program to increase the number of births occurring in health facilities. JSY improved the proportion of facility-based births amongst women of low socioeconomic status in rural areas [6], but had no impact on maternal mortality, largely because these rural primary health clinics (PHCs) lacked skilled birth attendants trained in evidence-based practices (EBP) [7]. Previous studies, such as the Community Level Interventions study for Preeclampsia (CLIP) in Karnataka have explored the capacity of healthcare providers to manage PE/E in Indian primary care settings. CLIP found that, while nurses and community health workers were familiar with the clinical severity of PE/E, large knowledge gaps existed regarding disease etiology and medication route and dosage [8]. In Bihar, a poor and largely rural state in northeastern India, these challenges are likely more severe [9].

Simulation-based training has been shown to promote use of EBPs in emergency obstetric care in low-resource settings, though use of specific skills varied [10–13]. However, the impact of simulation training on use of EBPs for diagnosis and management of PE/E in low-resource settings has not been reported. In order to be effective in this context, interventions must consider baseline knowledge and skills of providers [14, 15], as well as challenges inherent in magnesium sulfate and antihypertensive administration and continuous monitoring of maternal blood pressure (BP) [16, 17].

PRONTO International [18] developed a simulation-based training program to help address the need for provider training in emergency obstetric and neonatal care, including PE/E diagnosis and management, in Bihar. Simulation training was embedded within AMANAT, a large-scale nurse mentoring program developed by CARE India [19] and the Government of Bihar, and implemented at 320 PHCs across Bihar between 2015 and 2017. This study aimed to assess the impact of simulation-based training on PE/E diagnosis and management in Bihar by evaluating changes in nurse mentees’ use of EBPs in simulated PE/E cases and exploring perceived barriers and enablers of high quality PE/E care among nurse mentors.

## Methods

### Study design

This was a mixed methods study including quantitative and qualitative evaluations.

### Study setting

Bihar has a population of over 100 million, of which 89% is rural [20]. The maternal mortality rate (MMR) is 208 per 100,000 live births in Bihar, compared to 167 per 100,000 for India as a whole. In Bihar, each PHC serves an average population of ~ 190,000 [20], and one nurse midwife is frequently responsible for all obstetric emergency and delivery care at a given PHC.

### AMANAT training

The AMANAT nurse mentoring program was implemented in Bihar over four phases between March 2015 and January 2017. Each eight-month phase included 80 PHCs. A total of 120 college-educated nurse mentors, recruited from across India, participated in the program. Mentors completed four weeks of training with CARE India prior to beginning the program, including one week focused on simulation facilitation and debriefing that was conducted by PRONTO International. This was followed by a four-day advanced simulation facilitation course four months later. Mentees were nurses working at PHCs who were qualified in either Auxiliary Nurse Midwifery (ANM) or General Nursing and Midwifery (GNM), requiring 18 months and 3 years of nursing training, respectively, following completion of secondary school. Six to eight nurses at each PHC were selected to participate in the AMANAT program. Across the four phases, a total of 3422 mentees were trained statewide. Through AMANAT, mentees received training in Basic Emergency Obstetric and Neonatal Care [21].

During each phase, 40 nurse mentors rotated in pairs between four PHCs, visiting each PHC for one week per month. During each visit, mentors facilitated simulations of emergency obstetric and neonatal care scenarios, all of which were video-recorded. Each simulation was followed by a video-guided debrief, led by the nurse mentor. During debriefs, mentees were encouraged to reflect on simulations and consider how to apply what they learned to clinical practice. The curriculum included a total of 31 simulation scenarios. During week four of each phase, mentees received training on the key aspects of PE/E diagnosis and management through lectures, skills stations, and simulations. If time-permitted, mentors provided additional PE/E training during weeks five through eight.

### Part 1: Evaluating changes in nurse mentees’ use of EBPs in simulated PE/E cases

We evaluated changes in nurse mentees’ use of EBPs during simulated PE/E cases across all phases of the AMANAT program. The local PHC protocol for management of PE/E followed American College of Obstetricians and Gynecologists (2013) guidelines [22]. The expectation was that blood pressure (BP) be measured in all patients; if the BP was elevated (> 140/90) or if the patient complained of headache, guidelines advised taking a targeted history, performing a physical exam, and checking urine protein. Preeclampsia with severe features was defined as BP > 140/90 and any of the following: proteinuria (1+ or greater), new onset cerebral or visual disturbances, severe right upper quadrant or epigastric pain unaccountable by other diagnoses, systolic BP > 160, diastolic BP > 110. It is recommended that such patients be treated with a loading dose of magnesium sulfate (10 g IM and 4 g IV), an antihypertensive (usually nifedipine) if BP is in the severe range, targeting 130–140/100–90, and a foley catheter to monitor urine output. Next, depending on gestational age and stage of labor, the mother should be delivered in the clinic or referred to higher level of care. Eclamptic mothers require the same management with two additional steps, repositioning of mother to protect the airway onto her side and oxygen administration. PHCs in Bihar do not have the capacity to check protein/creatinine ratios, liver function tests, or platelet counts or preform cesarean deliveries. The PRONTO curriculum included two PE/E simulation scenarios, both of which involved a 17-year-old woman complaining of severe headache. If checked, mentees learned the patient had a BP of 170/112 with 3+ (brisk) reflexes, 2+ bilateral edema, and 3+ urine protein. In the second scenario, the woman progressed to have an eclamptic seizure after a few minutes. Simulation videos were matched by scenario type (e.g., preeclampsia with severe features or eclampsia) and by facility. PHCs with two or more videos of the same scenario were included unless two videos occurred on the same day. If three videos were available, the first and last completed videos were selected for inclusion in the analysis.

**Fig. 1:**
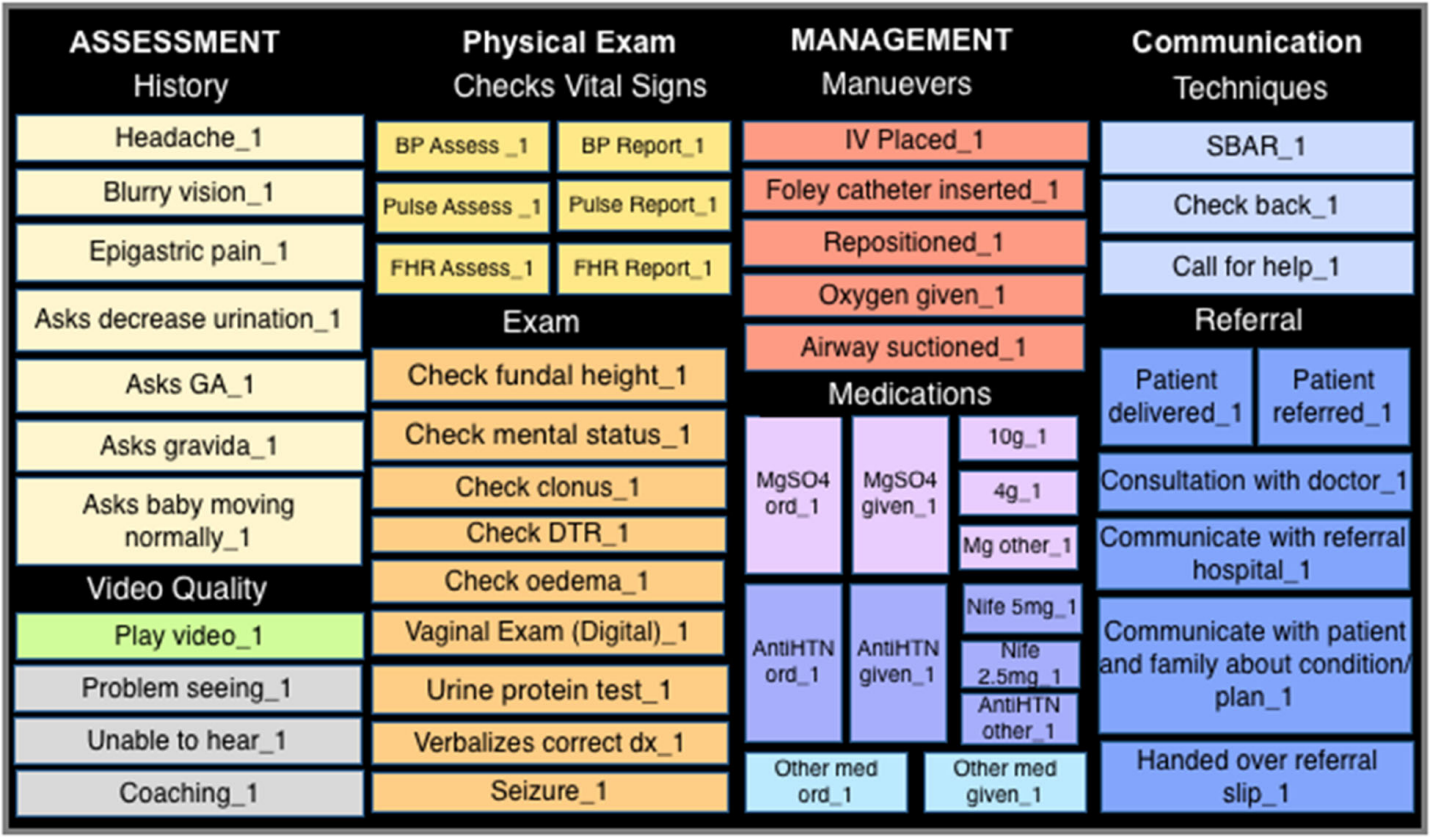
Severe Preeclampsia and Eclampsia Clinical Coding Window

EBP indicators were selected by clinical simulation experts from UCSF, PRONTO International, and CARE India. The indicators were used to develop a video analysis code window using Studiocode™ software (Fig. 1). Simulation videos were coded by two Hindi-speaking nurses in Patna, Bihar. Any indicators deemed to represent simulation artifact were excluded from the analysis.

Binary indictors were categorized by subgroup (Table 1), with composite scores calculated for each subgroup. Two continuous indicators assessed key time intervals: 1) ‘time from BP measurement to magnesium sulfate given,’ 2) ‘time from BP measurement to antihypertensive given.’

**Table 1:** Subgroups of binary indicators used to assess simulation videos

Subgroup	Scenario assessed
1) History questions (headache, blurry vision, epigastric pain, gestational age)	Both
2) Diagnostic tests [BP, heart rate (HR), fetal heart rate (FHR), clonus or deep tendon reflexes (DTR), edema, urine protein]	Both
3) Management steps (intravenous catheter placed, foley catheter inserted**,** magnesium sulfate given, antihypertensive given)	Both
4) Airway management steps (oxygen administered, patient repositioned laterally)	Eclampsia only

Sixteen videos (20.5%) were randomly selected for double coding to assess inter-rater reliability across the two coders. Cohen’s kappa was > 0.6 for all binary variables [23] except epigastric pain (kappa = 0.59) and ICC was > 0.9 for both continuous variables [24], demonstrating strong inter-rater reliability.

#### Statistical analysis

The proportion of EBPs completed, subgroup composite scores, and key time intervals during mentees’ first and last participation in PE/E simulations were compared using generalized estimating equations (GEE) to appropriately estimate standard errors, adjusted for time (in days). All analyses were conducted in R Core Team, version 0.99.903 [25].

### Part 2: Exploring perceived barriers and enablers of high quality PE/E care among nurse mentors

We explored barriers and enablers of high quality PE/E care through semi-structured interviews with current nurse mentors. Interviews were conducted in April 2017 in Patna. Interviewees were purposively selected, with preference given to mentors who had worked in different regions of Bihar as well as to those who had not previously participated in other interviews related to the AMANAT intervention. If both mentors working in a pair met these criteria, one was randomly selected for participation.

The interview guide employed open-ended questions and interviewers had flexibility to address emerging themes. Interviews were conducted by the first author and a Patna-based member of the PRONTO team. The Patna-based interviewer was fluent in Hindi and had qualitative research experience. All interviews were conducted in English. Interviews were held in private rooms at the PRONTO office or in private hotel rooms in Patna. Interview duration ranged from 42 to 66 min.

#### Thematic analysis

Interviews were transcribed by the first author, with assistance from a transcription service in Bihar. To improve transcription quality, the first author listened to audio recordings and revised transcriptions as needed. Data were analyzed using the thematic content approach, consisting of four steps: 1) data familiarization, 2) identifying codes and themes, 3) developing a coding scheme and applying it to the data, and 4) organizing codes and themes [26, 27]. Two interviews were double coded. Any discrepancies were discussed and resolved to develop the final coding framework, which was applied to all remaining transcripts.

### Ethical considerations

Written informed consent was obtained from all participants prior to the interview. Ethical approval was granted from the Committee on Human Research at the University of California San Francisco (14–15,446) and the Institutional Review Board of the Indian Institute of Health Management Research.

## Results

### Part 1: Evaluating changes in nurse mentees’ use of EBPs in simulated PE/E cases

A total of 39 paired simulation videos were analyzed. Simulations averaged 10 min (range: 2–33 min) and had a median of two participants each. The median duration between first and last participation in simulations was 62 days (range: 1–125 days).

The proportion of simulated PE/E cases in which mentees completed key clinical indicators are displayed in Table 2. The proportion of simulations in which mentees ‘asked about epigastric pain’ increased from 43.6 to 51.3% (*p* = 0.03) and the proportion in which ‘Foley catheter was inserted’ trended upwards from 38.5 to 56.4% (*p* = 0.06). The total number of ‘history questions asked’ increased from 1 to 2 (p = 0.03), and the total number of ‘management steps completed’ increased from 2 to 3 (*p* = 0.03).

**Table 2:** Proportion of simulated preeclampsia and eclampsia cases in which nurse mentees completed key history, diagnostic, and management steps (*N* = 39 matched pairs)

	First simulation	Last simulation	
History questions	*n* (%)^§^		*p*-value
Headache	28 (71.8)	32 (82.1)	0.25^‡^
Blurry vision	17 (43.6)	20 (51.3)	0.49^‡^
Epigastric pain	1 (2.6)	7 (17.9)	0.01^‡^
Asks gestational age	8 (20.5)	12 (30.8)	0.14^‡^
Total steps completed (median, IQR^*^)	1.0 (1.0–2.0)	2.0 (1.0–2.0)	0.03^∞^
Diagnostic tests			
BP assessed	39 (100.0)	39 (100.0)	NA
FHR assessed	29 (74.4)	30 (76.9)	0.08^‡^
Fundal height measured	3 (7.7)	7 (17.9)	0.15^‡^
Clonus or DTR	9 (23.1)	6 (15.4)	0.44^‡^
Edema	8 (20.5)	12 (30.8)	0.30^‡^
Urine protein test	12 (30.8)	11 (28.2)	0.80^‡^
Total steps completed (median, IQR^*^)	3.0 (2.0–3.0)	3.0 (2.0–3.0)	0.39^∞^
Management steps			
Magnesium sulfate given	33 (86.4)	32 (82.1)	0.74^‡^
Antihypertensive given	22 (56.4)	28 (71.8)	0.20^‡^
Intravenous catheter placed	16 (41.0)	24 (61.5)	0.08^‡^
Foley catheter inserted	15 (38.5)	22 (56.4)	0.06^‡^
Total steps completed (median, IQR^*^)	2.0 (1.5–3.0)	3.0 (2.0–4.0)	0.03^∞^
Airway management steps (*N* = 13 matched pairs^^^)			
Oxygen given	9 (34.6)	9 (34.6)	NA
Patient repositioned	19 (73.1)	17 (65.4)	0.56^‡^
Total steps completed (median, IQR^*^)	1.0 (0.25–2.0)	1.0 (0.25–1.75)	0.71^∞^

^§^*n* = Frequency of first and last simulated cases in which mentees completed key EBPs

% = Proportion of first and last simulated cases in which mentees completed key EBPs

^*^IQR = interquartile range of total number of steps completed

^#^Difference in proportion of EBPs completed from first to last participation in simulated case

^‡^GEE logistic regression adjusted for duration (in days) between first and last simulations

^∞^GEE linear regression adjusted for duration (in days) between first and last simulations

^^^Airway management steps analyzed in simulated eclampsia cases only

Time required for nurse mentees to complete key management steps in simulated PE/E cases is displayed in Table 3. Time from ‘BP measured to magnesium sulfate given’ remained relatively constant (*p* = 0.69), while time from ‘BP measured to antihypertensive given’ decreased by 3.1 min (*p* = 0.06) between first and last participation in PE/E simulations.

**Table 3:** Time to completion of key management steps by nurse mentees in simulated preeclampsia and eclampsia cases

		First simulation	Last simulation	
Time to completion of management steps	*n*	Median time in minutes (IQR)		*p*-value^‡^
BP measured to magnesium sulfate given	63	3.7 (2.2–4.5)	3.0 (1.8–6.4)	0.69
BP measured to antihypertensive given	47	5.8 (2.6–9.7)	2.6 (1.0–6.6)	0.06

^‡^GEE linear regression adjusted for duration (in days) between first and last simulations

### Part 2: Exploring perceived barriers and enablers of high quality PE/E care among nurse mentors

Twelve nurse mentors, with a median age of 25.5 years, participated in interviews. Interviewees had worked as mentors for a median of 1.5 years. They came from eight different Indian states: Uttar Pradesh (3), Mumbai (2), Bombay (1), Kerala (2), Delhi (2), Chhattisgarh (1), West Bengal (1). Notably, no mentors were from Bihar.

We used the main themes emerging from the data to structure the presentation of material from the interviews, with themes broadly classified as barriers or enablers, as detailed below.

### Barriers

#### Knowledge gaps

Despite tremendous improvement, the majority of mentors noted that mentees struggled to understand diagnostic criteria of preeclampsia vs preeclampsia with severe features. Nurses had a hard time shifting from the previous categorization of mild and severe preeclampsia and, even though our training focused on the more recent criteria, many mentors themselves still referred to the older mild and severe categorization.

*“They can do eclampsia and preeclampsia. But they’re confusing like mild [preeclampsia without severe features] and severe …*. *sometimes previously I also confuse what I will do.”* (Age 26–30).

Confusion with diagnostic criteria was likely exacerbated by the fact that mentees at times had trouble assessing the quality of symptoms.

*“Epigastric [pain] they are not able to differentiate with labor pain.”* (Age unknown).

Calculating the loading dose of magnesium sulfate, which required conversion of percentages to grams, was also very challenging for mentees.

*“Mentees [with] ANM training, they don’t know what is mg [milligram], so it’s quite difficult.”* (Age 26–30).

Half of mentors acknowledged, however, that mentees were much more likely to treat preeclampsia with severe features with magnesium sulfate after the training, and only one said this was happening in her PHC prior to the training.

Additionally, half of mentors felt that mentees continued to have difficulty managing eclamptic seizures. They attributed this to fear and the low incidence of eclampsia.

#### Interpersonal issues

All mentors perceived the strict hierarchy between nurses and doctors as a significant barrier to high quality care. The majority also perceived that poor relationships between nurses and patients in PHCs were key barriers.

*“Yeah, they [nurses] are scared. If they tell something, also the doctor will say that, ‘You know more than me, you’re a doctor. You think that you are a doctor. You are not there to teach me.’”* (Age 26–30).

The majority of mentors reported that aggressive behavior by family members of patients sometimes prevented nurses from providing evidence-based care. Nurse mentors were unsure of what led to this aggressive behavior, but they discussed fear, lack of education, previous medical mistreatment, and limited understanding of medical care as important factors.

*“If anything happens, they’re beating us.”* (Age 26–30).

*“Actually, the thing is, more than the staff nurses, the patients’ attendants [relatives] are more nervous. And because of their nervousness–the sisters [nurses] and doctors, they get nervous on top of that... So, it becomes a clash between them– and then the fight begins.”* (Age 26–30).

#### Resource limitations

All mentors agreed that human resource shortages, including doctors and nurses, were a key barrier. One to two nurses often covered the entire PHC, including emergency care, vaccinations, and labor and delivery. Doctors were rarely present.

*“So, twenty, for twenty patients, only one sister [nurse] is there to check blood pressure and take delivery. Often, it’s very difficult … so identification, early identification is not possible.”* (Age 26–30).

*“Most of the times doctors are not available in the PHCs. They used to go for some meetings or some trainings … Or they go to their private clinics*.” (Age 21–25).

Mentors felt that shortages of medications and urine protein strips were the most important supply-related barriers to high quality PE/E care, and half described lack of ambulances as a key problem. The combination of ambulance shortages, costly private vehicles, and long distances between PHCs and referral hospitals made it nearly impossible to effectively refer patients who required a higher level of care. One mentor described how the lack of supplies in one PHC prevented adequate treatment of a woman with preeclampsia with severe features.

*“I was scared … Because now, mother, she is having [a] bad headache. [Elevated] blood pressure is there. No magnesium sulfate is there. No nifedipine is there... After one hour, she got eclampsia.”* (Age unknown).

The mother described above was subsequently transferred to a private clinic, where she delivered vaginally without receiving any medications to treat her condition. She recovered, but her baby died shortly after delivery.

### Enablers

#### Simulation training

All mentors agreed that simulation training was an important enabler of high quality care. The majority felt that mentoring during live cases helped develop mentees’ confidence, facilitating their ability to independently treat PE/E.

*“Simulation is very important. And by doing simulation they will learn, they will remember that for lifetime. Because in theory [didactics] they will write and they will after some days they will forgot. By doing simulation they are remembering– yeah once I had this case and I manage like that.”* (Age 21–25).

#### Communication between doctors and nurses

Several mentors described how effective communication between doctors and nurses facilitated high quality care. Clinical discussions provided a formal setting to discuss complicated cases and review evidence-based care guidelines, fostering teamwork and increasing institutional support for mentees. Further, this platform allowed mentees to demonstrate their clinical proficiency, and some mentors believed this helped reduce hierarchical issues between doctors and nurses. Mentors also discussed the value of the two-challenge rule, a communication technique for respectfully asserting disagreement with superiors when there is a patient safety concern.

*“Some mentees are doing [the] two challenge rule with doctors. ‘We can’t [only] give Lasix because we are not preventing the convulsions. And for the blood pressure, we have to give nifedipine.’”* (Age 21–25).

#### Physician leadership

Mentors felt that doctor buy-in was critical to programmatic success. They described how AMANAT workshops helped doctors improve their skills in leadership and clinical care, particularly regarding accurate provision of intravenous magnesium sulfate.

*“In PHC, [the] medical officer will stay at home and, in many emergencies, they will call, just call … but now they are coming, they are seeing, so mentees are having support now.”* (Age 21–25).

## Discussion

To reduce maternal mortality in Bihar, it is essential that primary health providers are able to effectively diagnose and manage PE/E. We found that mentees had improved composite scores in ‘history taking’ and ‘management steps;’ however, only one individual EBP significantly improved from first to last participation in simulated PE/E cases. The reason for this is likely multifactorial, encompassing need for additional training and resource limitations. For example, the low rates of urine protein assessment and oxygen administration in simulated cases may be partially attributed to actual supply shortages [28–30]. While the total number of ‘management steps’ completed by mentees increased, it is notable that magnesium sulfate administration did not improve. This finding is in contrast to previous evaluations of PE/E simulation training in high-resource settings [31, 32]. Nonetheless, the 76% rate of magnesium sulfate administration in simulated cases is much higher than that seen in the CLIP study, which found intravenous magnesium sulfate was never administered by nurses in PHCs [8]. This suggests that the initial simulation scenario may have overestimated mentees’ baseline skills, particularly as they had likely already gained basic knowledge and skills through preceding lectures and skills stations. The inability of the simulation data to fully capture mentees improved skills was further supported by mentors’ discussion of the impact of simulation learning on their mentees’ clinical confidence and skills. For example, while mentors acknowledged that some mentees still called their mentors with questions regarding magnesium sulfate dosing and clarifying whether severe features were present or not, mentors shared many stories of mentees independently administering magnesium sulfate to mothers with preeclampsia with severe features. All but one mentor agreed that this would not have happened before training. These findings suggest that PE/E simulations may have an important role to play in improving diagnosis and management. Further studies should explore how PE/E simulation training may translate into changes in clinical practice among providers.

This study found that knowledge gaps, resource limitations, and interpersonal-related issues were key barriers to high quality of PE/E care in Bihar. Previous studies in India and meta-analyses from low-resource settings have also identified deficient supplies [16, 29], shortages of doctors and nurses [14], poor referral transport systems [14], and hierarchical issues among care providers [33, 34]. This study additionally identified aggressive behavior toward nurses by family members of patients, leading to fear of retaliation for negative health outcomes, as an additional barrier to provision of evidence-based, compassionate care in Bihar. This lack of therapeutic alliance at times could lead to catastrophic results for mothers. Mentors described situations in which providers would decide not to treat with magnesium or families would decline referral to a higher level of care, both of which are potentially life-saving practices. Future PRONTO interventions hope to address these challenges by including a unit on respectful maternity care that incorporates skills needed to manage family expectations.

Key enablers included simulation training, effective provider communication, and physician leadership. Other studies in low-resource settings have also identified participatory learning approaches [35], teamwork among doctors and nurses [3, 32, 36], and effective leadership [13] as facilitators of improved obstetric and emergency care. We found that communication techniques, such as the two-challenge rule, and clinical discussions can improve communication between providers, findings which are supported by results of related studies in high-resource settings [37, 38]. This suggests that team-based, inter-professional training can be successful in hierarchical cultures within Asia [39]. Finally, this study highlighted tailored workshops as an effective strategy to improve clinical and leadership skills among physicians, which in turn translated to higher motivation among mentees. This result parallels with studies from Kenya and South Africa that found that support from leadership was a key motivator for health workers and improved program success [40, 41].

This study has several limitations. Changes in mentees’ use of EBPs were evaluated by comparing their first and last participation in simulated PE/E cases. As a result of this approach, different amounts of time elapsed between simulations for different facilities. However, changes in mentee performance were robust to adjustments for time. The first simulated scenario may not represent a true baseline for mentees’ knowledge of PE/E, as the curriculum formally introduced preeclampsia in week 3 but mentees could have learned about it earlier if an affected patient had presented in week 1 or 2. Further, mentors gave lectures and facilitated rapid reviews prior to the first simulation in order to maximize mentee learning during simulations. Interviewers were members of the PRONTO team who were involved in training mentors, which may have facilitated social desirability bias. To increase content validity, a local Hindi interviewer was present at all interviews and participants were ensured their responses were completely confidential in nature. The quantitative results on use of EBPs were based on simulated scenarios, and generalizability of these findings to actual clinical practice is unknown.

## Conclusion

Simulation training improved mentees’ use of EBPs in simulated PE/E cases in Bihar, a key step to improving maternal survival. Knowledge gaps, resource limitations, and provider interpersonal issues were key barriers to PE/E care in PHCs. Simulation training, effective communication, and physician leadership were key enablers. The next iteration of the training curriculum will incorporate lessons learned from these findings. Notably, addressing resource-related barriers requires both financial support and political will. An improved understanding of key barriers and enablers of high quality PE/E care is an important initial step toward the design of contextually-targeted interventions to improve maternal survival in India.

## Abbreviations

ANM: Auxiliary Nurse Midwifery
BP: Blood pressure
CLIP: Community Level Interventions study for Preeclampsia
DTR: Deep tendon reflexes
EBP: Evidence-based practices
FHR: Fetal heart rate
GNM: General Nursing and Midwifery
JSY: Janani Suraksha Yojana
MMR: Maternal mortality rate
PE/E: Preeclampsia and eclampsia
PHC: Primary health clinics
